# A quest for sphincter-saving surgery in ultralow rectal tumours—a single-centre cohort study

**DOI:** 10.1186/s12957-018-1513-4

**Published:** 2018-11-07

**Authors:** Mateusz Rubinkiewicz, Piotr Zarzycki, Agata Czerwińska, Michał Wysocki, Natalia Gajewska, Grzegorz Torbicz, Andrzej Budzyński, Michał Pędziwiatr

**Affiliations:** 1 2nd Department of General Surgery, Jagiellonian University Medical College, Kopernika 21 Street, 31-501 Kraków, Poland; 2 Centre for Research, Training and Innovation in Surgery (CERTAIN Surgery), Kraków, Poland

**Keywords:** Intersphincteric resection, Transanal TME, Abdominoperineal resection, Rectal cancer

## Abstract

**Introduction:**

Despite the progress in the treatment of colorectal cancer, there is still no optimal strategy for tumours located adjacent to the anal sphincter. This study aims to evaluate oncological and functional results of surgery for rectal cancer in unfavourable locations in proximity to anal sphincters.

**Materials and methods:**

Patients with rectal cancer, which was either initially infiltrating the anal sphincter or located in the close proximity of the sphincter, were included in the study. Patients were submitted to extralevator abdominoperineal resection (APR), intersphincteric resection, or transanal total mesorectal excision (TaTME). Primary outcomes were perioperative data: operative time, blood loss, complications, length of stay (LOS), and 30-day mortality. Secondary outcomes were pathological quality of the specimens and functional outcome 6 months after defunctioning ileostomy closure.

**Results:**

Among patients with cancer adjacent to the anal sphincter, 13 (25%) underwent APR, 14 (27%) patients were submitted to intersphincteric resection, and 25 (48%) patients were treated with the TaTME approach. Operative time was 240 (210–270 IQR) for APR, 212.5 (170–260 IQR) for intersphincteric resection, and 270 (240–330 IQR) for TaTME (*p* = 0.018). Perioperative morbidity was 31% for APR, 36% for intersphincteric resections, and 12% for the TaTME group (*p* = 0.181). Complete mesorectal excision was achieved in 92% of specimens in the TaTME group, 93% in intersphincteric resections, and 78% in the APR group (*p* = 0.72). Median circumferential resection margin in APR was 6 mm (4–7 IQR), in intersphincteric resections 7.5 mm (2.5–10 IQR), and in the TaTME group 4 mm (2.8–8 IQR). All patients after intersphincteric resections developed major low anterior resection syndrome (LARS). Four patients in the TaTME group developed minor LARS, and 21 had major LARS.

**Conclusion:**

Sphincter-saving rectal resections are a feasible alternative to APR with good clinical, pathological, and oncological outcomes. Intersphincteric resections and TaTME seem to be equal in terms of clinicopathological results. The functional outcome is yet to be investigated.

**Trial registration:**

The study was retrospectively registered in Thai Clinical Trials Registry (23-07-2018, ID TCTR20180724001).

## Introduction

Despite the progress in the treatment of colorectal cancer, there is still no optimal strategy for tumours located adjacent to the anal sphincter. Typically, cancers of the low rectum were submitted to abdominoperineal resection (APR). Even though APR is believed to give predictable oncological results, its acceptance among patients is very low because of permanent colostomy [1, 2]. Introduction of the intersphincteric resection was hoped to result in better quality of life (QoL), related to sphincter preservation without compromising oncological outcomes [3–6]. Overall, the procedure has failed to attract the necessary popularity and is performed only in selected centres. The recently developed hybrid approach to rectal pathology, referred to as TaTME (transanal total mesorectal excision), allows for an important extension of indications for sphincter-sparing operations. With some modifications, it can also be used for ultralow tumours as an interesting alternative to intersphincteric resections. Despite continuous attempts to decrease the number of APR procedures [7], its numbers remain high. Our department for several years now has been promoting modern strategies aimed at the reduction of the APR rate. This study aims to evaluate oncological and functional results of surgery for rectal cancer in unfavourable locations in proximity to anal sphincters [8].

## Material and methods

### Setting

A retrospective cohort study, using a prospectively collected database of patients submitted to minimally invasive operations of rectal cancer, was evaluated. We are a tertiary referral university minimally invasive surgery unit with the annual volume of 120 laparoscopic colorectal surgeries, including 50 laparoscopic/hybrid rectal cancer resections. Initially, patients with cancer infiltration of the anal sphincter were submitted to laparoscopic extralevator abdominoperineal resection. In 2013, minimally invasive intersphincteric resections were introduced. The TaTME procedure was implemented in 2015, and in 2017, we were approved as a COLOR III participating centre [8]. Since 2016, all procedures requiring internal sphincter removal are performed by TaTME technique. All procedures were performed by a surgeon with expert skills in laparoscopic colorectal procedures. Pathological specimens were assessed by an experienced pathologist, according to guidelines delivered by Quirke et al. [9, 10]. The work has been reported in line with the STROCSS criteria [11].

### Patients

Patients with rectal cancer, which was either initially infiltrating the anal sphincter or located in the close proximity of the sphincter, were included in the study. Patients with > T2 tumours and/or positive lymph nodes were submitted for neoadjuvant chemoradiotherapy in order to gain downstaging according to current guidelines [12]. Before the surgery, MRI restaging was performed in all patients. Those who had persistent infiltration on the external anal sphincter or levator muscles were submitted to APR. Other patients were submitted for either laparoscopic intersphincteric resection or recently to the modified TaTME procedure. The operative technique of the abdominal part is described elsewhere [13]. We used the extralevator approach [14] for abdominoperineal resection. The technique of intersphincteric rectal resection described by Rullier was used [15]. In this approach, we used Lone Star Self-Retaining Retractor® for proper exposition of perianal tissues. The specimens were extracted transanally, and coloanal anastomosis was performed with single absorbable braided sutures. The TaTME technique was performed as described elsewhere [16]. As a modification, we used the Karl Storz TEO platform, which enables very low dissection, starting almost at the anal verge. Whenever possible, we tried to perform only partial resection of the internal sphincter or preferably only mucosectomy of the anal canal. Defunctioning ileostomy was created in all cases of sphincter-saving procedures. The Enhanced Recovery After Surgery (ERAS) protocol was used in perioperative care in all patients [17, 18]. Patients were followed up every 3 months after surgery. Patients with stage III and high-risk stage II tumours (poor response for neoadjuvant treatment, angioinvasion, inadequate lymphadenectomy, perforation of the tumour) in pathological assessment of the specimen were submitted for additional adjuvant chemotherapy according to the ESMO guidelines [19].

### Measured outcomes

Primary outcomes were perioperative data: operative time, blood loss, complications, length of stay (LOS), 30-day mortality, and 30-day readmission rate. Secondary outcomes were pathological quality of the specimens, functional outcome 6 months after defunctioning ileostomy closure, and short- and mid-term oncological outcomes. Perioperative complications were assessed according to the Clavien-Dindo classification [20]. Serious complications were defined as Clavien-Dindo III–V. Pathological quality parameters were assessed as completeness of mesorectal excision (intact mesorectal fascia), circumferential resection margin (CRM), and distal resection margin (DRM). CRM and DRM > 1 mm were considered as negative. Tumour regression grade (TRG) was assessed according to the AJCC criteria [21]. For functional outcome, we used the Jorge-Wexner scale and LARS (low anterior resection syndrome) score [22, 23]. The evaluation was performed 6 months after defunctioning ileostomy reversal in all patients. Patients were evaluated every 3 months post-surgery in order to detect distant metastases or local recurrence. The follow-up included colonoscopy and CT scan every year. Local recurrence was defined as any recurrence in the pelvic region. Distant metastases were defined as any recurrence outside the pelvis.

### Statistical analysis and ethical approval

All data were analysed with Statistica version 13.0 PL (StatSoft Inc., Tulsa, OK, USA). The results are presented as mean and standard deviation (SD), median, and interquartile range (IQR). The chi-square test of independence was used for evaluating categorical variables. The Shapiro-Wilk test was used to check for normal distribution of data, and the Student *t* test was used for normally distributed quantitative data. For non-normally distributed quantitative variables, the Mann-Whitney *U* test was used. *P* value < 0.05 was considered statistically significant.

All procedures were performed in accordance with the ethical standards of the 1964 Declaration of Helsinki and its later amendments (Fortaleza 2013). The study was approved by the Local Ethics Committee (No. 122.6120.198.2016). Every patient signed an informed consent prior to inclusion in the study.

## Results

**Fig. 1:**
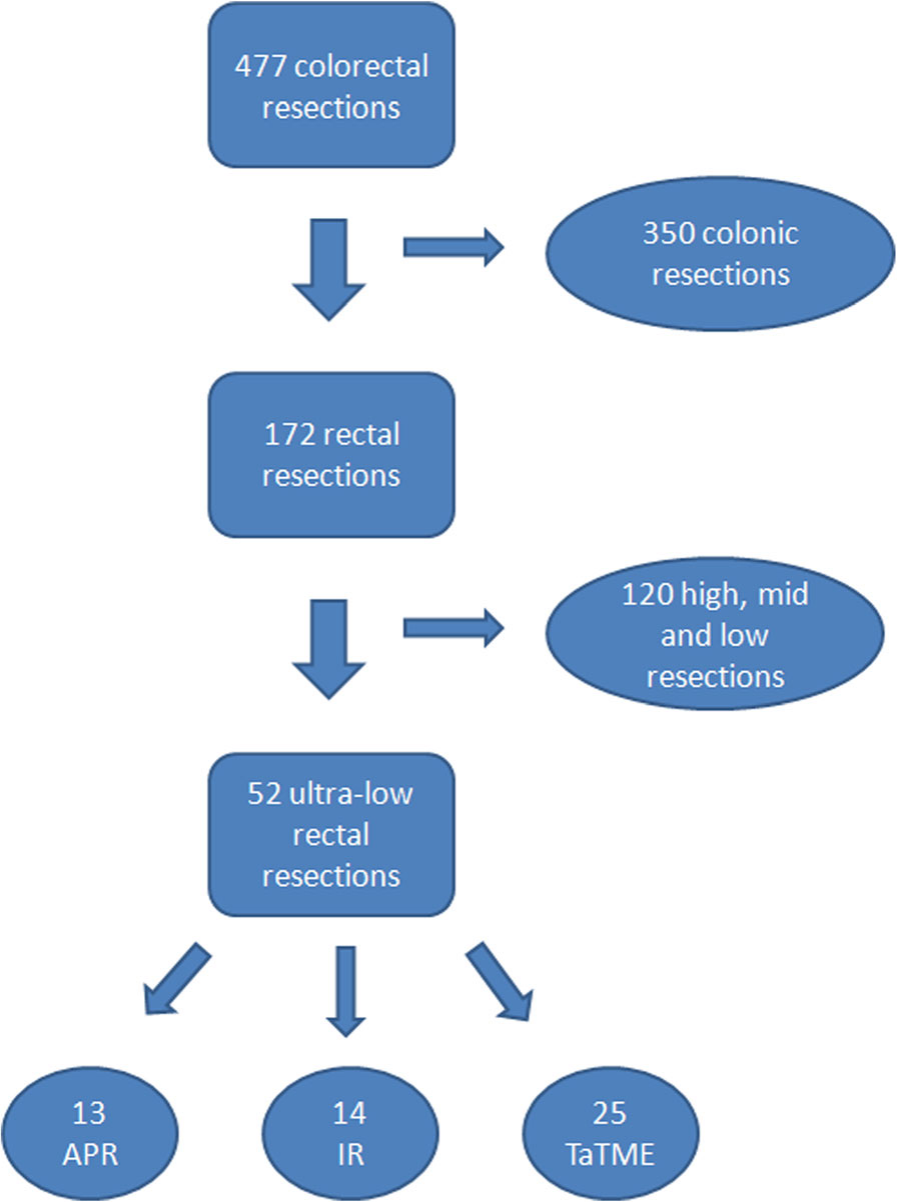
Study group design (APR—abdominoperineal resection, IR—intersphincteric resection, TaTME—transanal total mesorectal excision)

In the analysed period, 477 colorectal procedures were performed, including 172 rectal resections and 52 ultralow rectal resections. Among patients with cancer adjacent to the anal sphincter, 13 (25%) underwent APR, most of them (9 cases) between 2012 and 2015. Fourteen (27%) patients were submitted to intersphincteric resection. Twenty-five (48%) patients were treated with the modified TaTME approach. The study group design is presented in Fig. 1. Group characteristics are presented in Table 1. Tumours were localised at median 2 cm (range 1–4) from the anal verge for APR patients, 2 cm (range 1–5) for intersphincteric resections, and 2 cm (range 1–4) for TaTME patients (*p* = 0.21). Among patients treated with APR, 2 had infiltration on the internal sphincter and were treated before the introduction of intersphincteric resection, 5 had persistent infiltration on the external anal sphincter, and 4 had infiltration on levator muscles. Three patients from the TaTME group had distant metastases to the liver, which were resectable and were removed in the second stage. Two patients underwent laparoscopic metastasectomy, and 1 underwent laparoscopic right hemihepatectomy.

**Table 1:** Groups’ characteristics

	APR	Intersphincteric	TaTME	*p* value
Males/females, *n* (%)	8/5 (52%/48%)	12/2 (86%/14%)	19/6 (76%/24%)	0.345
Median age, years (IQR)	60 (55–71)	63 (50–69)	65 (59.5–72.5)	0.462
Median BMI, kg/m^2^ (IQR)	24.91 (22.31–27.40)	24.82 (24.15–28.73)	24.80 (21.94–26.47)	0.715
ASA class				
3	3 (23.08%)	6 (42.86%)	4 (16%)	0.175
2	10 (76.92%)	8 (57.14%)	21 (84%)	
T				
3	7 (53.85%)	6 (42.86%)	15 (60%)	0.311
2	4 (30.77%)	4 (28.57%)	1 (4%)	
1	1 (7.69%)	1 (7.14%)	2 (8%)	
0	1 (7.69%)	3 (21.43%)	7 (28%)	
N				
2	0	1 (7.14%)	5 (20%)	0.381
1	2 (15.38%)	3 (21.43%)	5 (20%	
0	11 (84.62%)	10 (71.43%)	15 (60%)	
M				
1	0	0	3 (12%)	0.179
0	13 (100%)	14 (100%)	22 (88%)	
AJCC				
4	0	0	4 (16%)	0.414
3	4 (30.77%)	6 (42.86%)	6 (24%)	
2	7 (53.85%)	5 (35.71%)	7 (28%)	
1	1 (7.69%)	1 (7.14%)	4 (16%)	
0	1 (7.69%)	2 (14.29%)	4 (16%)	
Neoadjuvant treatment, *n* (%)	11 (84.62%)	13 (92.86%)	24 (96%)	0.456
Adjuvant treatment, *n* (%)	6 (46%)	7 (50%)	13(52%)	0.943
Tumour regression grade (TRG)				
3	1 (7.69%)	2 (14.29%)	3 (12%)	0.949
2	7 (53.85%)	7 (50%)	14 (56%)	
1	4 (30.77%)	3 (21.43%)	4 (16%)	
0	1 (7.69%)	2 (14.29%)	4 (16%)	

*AJCC* American Joint Committee on Cancer staging system, *ASA* American Society of Anesthesiologists class, *BMI* Body Mass Index, *IQR* interquartile range, *T,N,M* TNM classification

Operative time was 240 (210–270 IQR) for APR, 212.5 (170–260 IQR) for intersphincteric resection, and 270 (240–330 IQR) for TaTME (*p* = 0.018). Perioperative outcomes are presented in Table 2. Perioperative morbidity was 31% for APR, 36% for intersphincteric resections, and 12% for the TaTME group (*p* = 0.181). Serious complications occurred in 2 patients in the intersphincteric group and in 2 patients in the TaTME group. Complete mesorectal excision was achieved in 92% of specimens in the TaTME group, 93% in the intersphincteric resections, and 78% in the APR group (*p* = 0.72). In other cases, mesorectal excision was nearly complete (< 5 mm of damage on the mesorectal fascia). CRM was negative in all cases. Median CRM in APR was 6 mm (4–7 IQR), in intersphincteric resections 7.5 mm (2.5–10 IQR), and in the TaTME group 4 mm (2.8–8 IQR). We had 2 cases of margin < 1 mm in TaTME patients and 1 in the APR group.

**Table 2:** Primary outcomes

	APR	Intersphincteric	TaTME	*p* value
Median operative time, min (IQR)	240 (210–270)	212.5 (170–260)	270 (240–330)	0.018
Median blood loss, ml (IQR)	125 (100–200)	150 (50–200)	150 (100–200)	0.856
Intraoperative adverse events (IAE), *n* (%)	1 (7.69%)	1 (7.14%)	1 (4%)	0.869
Anastomosis				
Circular stapled	0	1 (7.14%)	19 (76%)	< 0.001
Hand-sewn coloanal	0	13 (92.86%)	6 (24%)	
No anastomosis	13 (100%)	0	0	
Sphincter excision (no excision/internal/external) (no. of patients)	0/0/13	1/12/0	20/5/0	< 0.001
Ileostomy, *n* (%)	0	14 (100%)	25 (100%)	< 0.001
Perioperative morbidity	4 (30.77%)	5 (35.71%)	3 (12%)	0.181
Perioperative mortality	0	0	0	n/a
Clavien-Dindo				
III	0	2	2	0.094
II	4	3	1	

All patients after intersphincteric resections developed major LARS with median 34 (32–34 IQR) LARS score and 12 (11–14 IQR) points in Wexner scale. Four patients in the TaTME group developed minor LARS, and 21 had major LARS. Median LARS score for the whole TaTME group was 32 (30–37 IQR) and 11 (8–12 IQR) in Wexner scale. One patient in the TaTME group had to have permanent colostomy created due to perioperative complications, and 1 patient in the intersphincteric group undergone end colostomy creation due to inability to accept LARS syndrome.

After the 1-year follow-up, 85% of patients with APR reached disease-free survival (DFS), whereas DFS was 92% in the intersphincteric group and 96% in the TaTME group (*p* = 0.86). One-year overall survival (OS) was 100% in the TaTME group, 100% in the intersphincteric group, and 93% in the APR group. All patients completed the 1-year follow-up. The summary of secondary outcomes is placed in Table 3.

**Table 3:** Secondary outcomes

	APR	Intersphincteric	TaTME	*p* value
Median CRM, mm (IQR)	6 (4–7)	7.5 (2.5–10)	4 (2.8–8)	0.682
Median DRM, mm (IQR)	28 (12–35)	11 (5–15)	10 (7–15)	0.008
Preoperative Wexner	n/a	2 (1–2)	0 (0–2)	0.165
Postoperative Wexner	n/a	12 (11–14)	11 (8–12)	0.475
Preoperative LARS	n/a	4 (4–6)	5 (0–12)	0.999
Postoperative LARS	n/a	34 (32–34)	32 (30–37)	0.397

## Discussion

In the course of 5 years, we were able to perform sphincter-saving procedures in 75% of patients. In 25% of our patients, we performed APR which was inevitable in those cases. Unfortunately, the majority of patients suffered from mild to major LARS symptoms, both in the intersphincteric and TaTME group. We were unable to document superiority of any of those approaches in terms of functional results.

APR has been considered a procedure of choice for very low rectal cancer to achieve a negative resection margin. Initially, the technique was suboptimal leading to a high rate of positive margins and perforations in cases of low tumours frequently adjacent to pelvic floor muscles. The introduction of extralevator APR improved the outcomes, but APR remained one of the most stigmatising procedures in abdominal surgery. This was until sphincter-saving procedures were introduced. The shift from extensive operations such as APR was possible thanks to several reasons. The introduction of the minimally invasive approach and preoperative neoadjuvant therapy plays a crucial role in the matter in question. Laparoscopy and transanal videoendoscopy facilitated better visualisation of the operative field. The magnified view allows for more precise identification of tissue layers, which results in wider resection margins and a lower rate of local recurrence, which was confirmed in COLOR II—a large randomised multicentre study—and also in the meta-analysis of available trials [24–26]. Better visualisation may also help to spare pelvic nerves, whose damage is postulated as a possible cause of faecal incontinence [27, 28]. Rullier et al. observed that the treatment of tumours of the anorectal junction through the combination of preoperative radiotherapy with sphincter-saving procedures not only may facilitate good perioperative and functional outcomes, but is also safe from the oncological point of view [29]. His classical approach without videoendoscopy was possible thanks to new devices for perineal step of rectal resection like the Lone Star retractor. The system provides extraordinary visualisation of the perianal region, which is much better than that provided by other retractors or anal retraction sutures, and this enables the surgeon to focus on the quality of surgery [30]. With this advances, open and laparoscopic intersphincteric rectal resection occurred to be procedures with acceptable clinical outcome [31]. However, the popularity of this approach remains surprisingly low. It is certainly more demanding than APR, and functional results may look disappointing, but on the other hand, there is a strong demand among patients for sphincter preservation. The recently introduced TaTME is receiving more interest and acceptance among surgeons. This technique with some modifications may allow for ultralow resection and saving of the sphincter. This is possible due to the extraordinary exposure of the operating field, which facilitates more precise surgical techniques.

Neoadjuvant chemoradiotherapy plays an indisputable role in minimising the aggressiveness of surgical procedures. Those methods result in significant downstaging of rectal tumours, including T4 cancer [32, 33]. For ultralow cancer, neoadjuvant radiotherapy provides an additional benefit in extending the chance for sphincter-saving procedures [34]. Moreover, complete pathological response to radiotherapy is associated with a lower local recurrence rate and improved overall and disease-free survival [35, 36]. In our material, nearly 90% of patients responded to neoadjuvant chemoradiotherapy; however, only 13% had complete pathological response. Nevertheless, the response to radiotherapy was considered in our series as an indication for sphincter-saving procedures, as the “wait and watch” approach still requires full evaluation.

We obtained satisfactory outcomes regarding clinical features and the quality of pathological specimens using the intersphincteric resection technique. What is worth mentioning is that all our patients were treated with high compliance to the ERAS protocol, which improved clinical results in abdominal surgery [18, 25, 37, 38]. Our experiences confirm the results of Laurent et al. which state that laparoscopic intersphincteric rectal resection gives acceptable clinical outcomes [31]. Moreover, intersphincteric resection provides good oncological outcomes, comparable to APR [39–41]. Also, TaTME procedures yield satisfactory results concerning the quality of pathological specimens with a proper resection margin. When comparing clinicopathological features in our material, we failed to demonstrate the superiority of TaTME over intersphincteric resection; however, our subjective opinion resulted in an almost complete shift to the TaTME procedure in ultralow tumours. We had 2 patients with a resection margin < 1 mm in the TaTME group; thus, some comment in this field is necessary. We failed to show any local recurrence during our follow-up in these patients. This is in line with the observations of others. Fitzgerald et al. report that in surgical treatment combined with radiotherapy, the completeness of mesorectal excision and the negative resection margin (regardless of the distance) are the most important factors for oncological outcomes [42]. Moreover, an up-to-date secondary analysis of CAO/ARO/AIO-04 trial underlines the association between the quality of mesorectal excision and the 3-year overall and disease-free survival, which is more important than the length of the margin [43].

All patients with sphincter-saving procedures, both in the TaTME and intersphincteric group, developed faecal incontinence of different severity. However, only 1 patient from our material who underwent intersphincteric resection demanded a conversion to a permanent stoma due to severe faecal incontinence, this being an argument supporting the wide use of sphincter-saving procedures. Others, despite various degrees of incontinence, were satisfied with the result and refused colostomy. The high values of LARS and Wexner scores in both groups of sphincter-saving procedures in our study may result from the relatively short (6-month) follow-up period, as most authors agree that the bowel and preserved sphincter require more time for proper adaptation. Nonetheless, the functional outcomes seem to be promising with high QoL [44]. Kupsch et al. also obtained good results regarding faecal continence; however, median time of evaluation was 6.5 years after stoma closure [45]. Chen et al. in their analysis reaching median 14.6 years of follow-up noted 46% of major LARS in their population [46]. Nowakowski et al. also report that defunctioning ileostomy, which is a standard procedure in sphincter-saving procedures, may contribute to LARS development [47]. Moreover, Celerier observed 22% of necessity for definitive stoma formation in a 10-year observation period [48]. Thus, to fully evaluate the outcomes in our material, we currently follow up our patients to receive more data and perhaps detect differences in faecal continence by comparing the TaTME and intersphincteric group. As the procedure is new, its functional outcomes are not yet well investigated; however, faecal continence assessment is a part of running RCTs as COLOR III protocol [8]. According to our knowledge, there are no up-to-date studies which compare the directly functional outcome in TaTME and intersphincteric procedures. In our series, the TaTME patients seem to control defecation better than patients undergoing intersphincteric resection; however, our study population is rather low and the follow-up period is too short to draw definite conclusions. The full evaluation of the functional outcomes of rectal resections is still a matter of further consideration.

High-quality randomised control trials comparing sphincter-saving procedures with abdominoperineal resections are non-existent and probably impossible to perform. Therefore, the evidence based on a prospective evaluation of results of sphincter-sparing surgery is crucial to justify the applicability of non-APR procedures in low rectal cancer.

Our study has some limitations. Firstly, this is a single-centre study with a limited number of cases. However, the procedures analysed demand expert skills in colorectal surgery; thus, recruiting other centres is difficult. Also, we were able to analyse only 1-year overall and disease-free survival. Although the initial procedures were performed in 2013, TaTME was introduced in 2015; thus, only few patients completed a 3-year observational period. Therefore, we resigned from presenting incomplete data in order to avoid bias due to different time of follow-up. We also did not analyse other types of surgery factors that may contribute to LARS development, for example, the type of radiotherapy [49].

## Conclusion

Sphincter-saving rectal resections are a feasible alternative to APR with good clinical, pathological, and oncological outcomes. Intersphincteric resections and TaTME seem to be equal in terms of clinicopathological results. The functional outcome is yet to be investigated. Efforts should be taken to limit the indications for APR.

## Abbreviations

APR: Abdominoperineal resection
CRM: Circumferential resection margin
DRM: Distal resection margin
ERAS: Enhanced Recovery After Surgery
IQR: Interquartile range
LARS: Low anterior resection syndrome
LOS: Length of stay
QoL: Quality of life
SD: Standard deviation
TaTME: Transanal total mesorectal excision

## References

[1] Konanz J, Herrle F, Weiss C, Post S, Kienle P (2013). Quality of life of patients after low anterior, intersphincteric, and abdominoperineal resection for rectal cancer—a matched-pair analysis. Int J Color Dis.

[2] How P, Shihab O, Tekkis P, Brown G, Quirke P, Heald R, Moran B (2011). A systematic review of cancer related patient outcomes after anterior resection and abdominoperineal excision for rectal cancer in the total mesorectal excision era. Surg Oncol.

[3] Schiessel R., Karner-Hanusch J., Herbst F., Teleky B., Wunderlich M. (1994). Intersphincteric resection for low rectal tumours. British Journal of Surgery.

[4] Molnar C, Vlad-Olimpiu B, Marian B, Cornelia T, Simona G (2018). Survival and functional and oncological outcomes following intersphincteric resection for low rectal cancer: short-term results. J Int Med Res.

[5] Okamura R, Hida K, Yamaguchi T, Akagi T, Konishi T, Yamamoto M, Ota M, Matoba S, Bando H, Goto S, Sakai Y, Watanabe M, Watanabe K, Otsuka K, Takemasa I, Tanaka K, Ikeda M, Matsuda C, Fukuda M, Hasegawa J, Akamoto S, Shiozawa M, Tsuruta A, Akiyoshi T, Kato T, Tsukamoto S, Ito M, Naito M, Kanazawa A, Takahashi T, Ueki T, Hayashi Y, Morita S, Yamaguchi T, Nakanishi M, Hasegawa H, Okamoto K, Teraishi F, Sumi Y, Tashiro J, Yatsuoka T, Nishimura Y, Okita K, Kobatake T, Horie H, Miyakura Y, Ro H, Nagakari K, Hidaka E, Umemoto T, Nishigori H, Murata K, Wakayama F, Makizumi R, Fujii S, Sunami E, Kobayashi H, Nakagawa R, Enomoto T, Ohnuma S, Higashijima J, Ozawa H, Ashida K, Fujita F, Uehara K, Maruyama S, Ohyama M, Yamamoto S, Hinoi T, Yoshimitsu M, Okajima M, Tanimura S, Kawasaki M, Ide Y, Hazama S, Watanabe J, Inagaki D, Toyokawa A, Japan Society of Laparoscopic Colorectal Surgery (2017). Local control of sphincter-preserving procedures and abdominoperineal resection for locally advanced low rectal cancer: propensity score matched analysis. Ann Gastroenterol Surg.

[6] Abou-Zeid AA, El Ghamrini Y, Youssef T (2015). The combined abdominal and perineal approach for dissection of the lower rectum. The development of new indications. Int J Surg.

[7] Engel AF, Oomen JLT, Eijsbouts QAJ, Cuesta MA, van de Velde CJH (2003). Nationwide decline in annual numbers of abdomino-perineal resections: effect of a successful national trial?. Color Dis.

[8] Deijen CL, Velthuis S, Tsai A, Mavroveli S, de Lange-de Klerk ESM, Sietses C, Tuynman JB, Lacy AM, Hanna GB, Bonjer HJ (2016). COLOR III: a multicentre randomised clinical trial comparing transanal TME versus laparoscopic TME for mid and low rectal cancer. Surg Endosc.

[9] Quirke P, Morris E (2007). Reporting colorectal cancer. Histopathology.

[10] Nagtegaal ID, van de Velde CJH, van der Worp E, Kapiteijn E, Quirke P, van Krieken JHJM, Cooperative clinical investigators of the Dutch colorectal Cancer Group (2002). Macroscopic evaluation of rectal cancer resection specimen: clinical significance of the pathologist in quality control. J Clin Oncol.

[11] Agha RA, Borrelli MR, Vella-Baldacchino M, Thavayogan R, Orgill DP, Pai PS, Basu S, McCaul J, Millham F, Vasudevan B, Leles CR, Rosin RD, Klappenbach R, Machado-Aranda DA, Perakath B, Beamish AJ, Thorat MA, Ather MH, Farooq N, Laskin DM, Raveendran K, Albrecht J, Milburn J, Miguel D, Mukherjee I, Valmasoni M, Ngu J, Kirshtein B, Raison N, Boscoe M, Johnston MJ, Hoffman J, Bashashati M, Thoma A, Healy D, Orgill DP, Giordano S, Muensterer OJ, Kadioglu H, Alsawadi A, Bradley PJ, Nixon IJ, Massarut S, Challacombe B, Noureldin A, Chalkoo M, Afifi RY, Agha RA, Aronson JK, Pidgeon TE, D. STROCSS Group (2017). The STROCSS statement: strengthening the reporting of cohort studies in surgery. Int J Surg.

[12] van de Velde CJH, Boelens PG, Borras JM, Coebergh J-W, Cervantes A, Blomqvist L, Beets-Tan RGH, van den Broek CBM, Brown G, Van Cutsem E, Espin E, Haustermans K, Glimelius B, Iversen LH, van Krieken JH, Marijnen CAM, Henning G, Gore-Booth J, Meldolesi E, Mroczkowski P, Nagtegaal I, Naredi P, Ortiz H, Påhlman L, Quirke P, Rödel C, Roth A, Rutten H, Schmoll HJ, Smith JJ, Tanis PJ, Taylor C, Wibe A, Wiggers T, Gambacorta MA, Aristei C, Valentini V (2014). EURECCA colorectal: multidisciplinary management: European consensus conference colon & rectum. Eur J Cancer.

[13] Wu Wen-Xi, Sun Yao-Min, Hua Yi-Bin, Shen Li-Zong (2004). Laparoscopic versus conventional open resection of rectal carcinoma: A clinical comparative study. World Journal of Gastroenterology.

[14] Holm T, Ljung A, Häggmark T, Jurell G, Lagergren J (2007). Extended abdominoperineal resection with gluteus maximus flap reconstruction of the pelvic floor for rectal cancer. Br J Surg.

[15] Rullier Eric, Laurent Christophe, Bretagnol Fr??d??ric, Rullier Anne, Vendrely V??ronique, Zerbib Frank (2005). Sphincter-Saving Resection for All Rectal Carcinomas. Annals of Surgery.

[16] Suwanabol P, Maykel J (2017). Transanal total mesorectal excision: a novel approach to rectal surgery. Clin Colon Rectal Surg.

[17] Pędziwiatr M, Pisarska M, Kisielewski M, Major P, Mydlowska A, Rubinkiewicz M, Winiarski M, Budzyński A. ERAS protocol in laparoscopic surgery for colonic versus rectal carcinoma: are there differences in short-term outcomes? Med Oncol. 2016;33. 10.1007/s12032-016-0772-6. 10.1007/s12032-016-0772-6 PMC4859853 27154634

[18] Kisielewski Michał, Rubinkiewicz Mateusz, Pędziwiatr Michał, Pisarska Magdalena, Migaczewski Marcin, Dembiński Marcin, Major Piotr, Rembiasz Kazimierz, Budzyński Andrzej (2017). Are we ready for the ERAS protocol in colorectal surgery?. Videosurgery and Other Miniinvasive Techniques.

[19] Glynne-Jones R, Wyrwicz L, Tiret E, Brown G, Rödel C, Cervantes A, Arnold D, ESMO Guidelines Committee (2017). Rectal cancer: ESMO Clinical Practice Guidelines for diagnosis, treatment and follow-up. Ann Oncol.

[20] Clavien PA, Barkun J, de Oliveira ML, Vauthey JN, Dindo D, Schulick RD, de Santibañes E, Pekolj J, Slankamenac K, Bassi C, Graf R, Vonlanthen R, Padbury R, Cameron JL, Makuuchi M (2009). The Clavien-Dindo classification of surgical complications: five-year experience. Ann Surg.

[21] Edge SB, Compton CC (2010). The American Joint Committee on Cancer: the 7th edition of the AJCC cancer staging manual and the future of TNM. Ann Surg Oncol.

[22] Jorge Marcio J. N., Wexner Steven D. (1993). Etiology and management of fecal incontinence. Diseases of the Colon & Rectum.

[23] Emmertsen KJ, Laurberg S (2012). Low anterior resection syndrome score. Ann Surg.

[24] Bonjer HJ, Deijen CL, Abis GA, Cuesta MA, van der Pas MHGM, de Lange-de Klerk ESM, Lacy AM, Bemelman WA, Andersson J, Angenete E, Rosenberg J, Fuerst A, Haglind E, COLOR II Study Group (2015). A randomized trial of laparoscopic versus open surgery for rectal cancer. N Engl J Med.

[25] Pędziwiatr M, Małczak P, Mizera M, Witowski J, Torbicz G, Major P, Pisarska M, Wysocki M, Budzyński A (2017). There is no difference in outcome between laparoscopic and open surgery for rectal cancer: a systematic review and meta-analysis on short- and long-term oncologic outcomes. Tech Coloproctol.

[26] P. Małczak, G.T. Magdalena Mizera, J. Witowski, P. Major, M. Pisarska, M. Wysocki, M. Strzałka, A. Budzyński, M. Pędziwiatr Is the laparoscopic approach for rectal cancer superior to open surgery? A systematic review and meta-analysis on short-term surgical outcomes., Wideochir. Inne Tech. Mało Inwazyjne. (2018) 1–12. 10.5114/wiitm.2018.75845 PMC6041579 30002744

[27] Nowakowski Michał, Tomaszewski Krzysztof A., Herman Roman M., Sałówka Jerzy, Romaniszyn Michał, Rubinkiewicz Mateusz, Walocha Jerzy A. (2014). Developing a new electromyography-based algorithm to diagnose the etiology of fecal incontinence. International Journal of Colorectal Disease.

[28] Reibetanz J, Kim M, Germer C, Schlegel N (2015). Late complications and functional disorders after rectal resection: prevention, detection and therapy. Chirurg.

[29] Rullier E, Laurent C, Zerbib F, Belleannée G, Caudry M, Saric J. Conservative treatment of adenocarcinomas of the anorectal junction by preoperative radiotherapy and intersphincteral resection. Ann Chir. 2000;125:618–24 http://www.ncbi.nlm.nih.gov/pubmed/11051690 (accessed 29 June 2018). 10.1016/s0003-3944(00)00262-5 11051690

[30] Durai R, Makhija R (2015). Anal retraction sutures as an alternative to Lone Star ® retractor. Ann R Coll Surg Engl.

[31] Laurent C, Paumet T, Leblanc F, Denost Q, Rullier E (2012). Intersphincteric resection for low rectal cancer: laparoscopic vs open surgery approach. Color Dis.

[32] Krishnamurty DM, Hawkins AT, Wells KO, Mutch MG, Silviera ML, Glasgow SC, Hunt SR, Dharmarajan S (2018). Neoadjuvant radiation therapy in locally advanced colon cancer: a cohort analysis. J Gastrointest Surg.

[33] Liu G-C, Yan J-P, He Q, An X, Pan Z-Z, Ding P-R (2016). Effect of neoadjuvant chemoradiotherapy with capecitabine versus fluorouracil for locally advanced rectal cancer: a meta-analysis. Gastroenterol Res Pract.

[34] Latkauskas T, Pauzas H, Gineikiene I, Janciauskiene R, Juozaityte E, Saladzinskas Z, Tamelis A, Pavalkis D (2012). Initial results of a randomized controlled trial comparing clinical and pathological downstaging of rectal cancer after preoperative short-course radiotherapy or long-term chemoradiotherapy, both with delayed surgery. Color Dis.

[35] Martin ST, Heneghan HM, Winter DC (2012). Systematic review and meta-analysis of outcomes following pathological complete response to neoadjuvant chemoradiotherapy for rectal cancer. Br J Surg.

[36] Zorcolo L, Rosman AS, Restivo A, Pisano M, Nigri GR, Fancellu A, Melis M (2012). Complete pathologic response after combined modality treatment for rectal cancer and long-term survival: a meta-analysis. Ann Surg Oncol.

[37] Pisarska M, Małczak P, Major P, Wysocki M, Budzyński A, Pędziwiatr M (2017). Enhanced recovery after surgery protocol in oesophageal cancer surgery: systematic review and meta-analysis. PLoS One.

[38] Małczak P, Pisarska M, Piotr M, Wysocki M, Budzyński A, Pędziwiatr M (2017). Enhanced recovery after bariatric surgery: systematic review and meta-analysis. Obes Surg.

[39] Goligher J. C., Dukes C. E., Bussey H. J. R. (1951). Local recurrences after sphincter-saving excisions for carcinoma of the rectum and rectosigmoid. British Journal of Surgery.

[40] Tulina IA, Bredikhin MI, Gerasimov AN, Krylov NN, Reshetov IV, Tsarkov PV. Intersphincteric resection for stage I-III low rectal cancer is an oncologically safe alternative to extralevator abdomino-perineal rectal resection. Khirurgiia (Sofiia). 2017:61–8 http://www.ncbi.nlm.nih.gov/pubmed/28418371 (accessed 27 June 2018). 10.17116/hirurgia2017461-68 28418371

[41] Abdel-Gawad W, Zaghloul A, Fakhr I, Sakr M, Shabana A, Lotayef M, Mansour O (2014). Evaluation of the frequency and pattern of local recurrence following intersphincteric resection for ultra-low rectal cancer. J Egypt Natl Canc Inst.

[42] Fitzgerald TL, Brinkley J, Zervos EE (2011). Pushing the envelope beyond a centimeter in rectal cancer: oncologic implications of close, but negative margins. J Am Coll Surg.

[43] Kitz Julia, Fokas Emmanouil, Beissbarth Tim, Ströbel Philipp, Wittekind Christian, Hartmann Arndt, Rüschoff Josef, Papadopoulos Thomas, Rösler Elisabeth, Ortloff-Kittredge Peter, Kania Ulrich, Schlitt Hans, Link Karl-Heinrich, Bechstein Wolf, Raab Hans-Rudolf, Staib Ludger, Germer Christoph-Thomas, Liersch Torsten, Sauer Rolf, Rödel Claus, Ghadimi Michael, Hohenberger Werner (2018). Association of Plane of Total Mesorectal Excision With Prognosis of Rectal Cancer. JAMA Surgery.

[44] Mahalingam S, Seshadri RA, Veeraiah S (2017). Long-term functional and oncological outcomes following intersphincteric resection for low rectal cancers. Indian J Surg Oncol.

[45] Kupsch J, Jackisch T, Matzel KE, Zimmer J, Schreiber A, Sims A, Witzigmann H, Stelzner S (2018). Outcome of bowel function following anterior resection for rectal cancer—an analysis using the low anterior resection syndrome (LARS) score. Int J Color Dis.

[46] Chen TY-T, Wiltink LM, Nout RA, Meershoek-Klein Kranenbarg E, Laurberg S, Marijnen CAM, van de Velde CJH (2015). Bowel function 14 years after preoperative short-course radiotherapy and total mesorectal excision for rectal cancer: report of a multicenter randomized trial. Clin Colorectal Cancer.

[47] Nowakowski Michał M., Rubinkiewicz Mateusz, Gajewska Natalia, Torbicz Grzegorz, Wysocki Michał, Małczak Piotr, Major Piotr, Wierdak Mateusz, Budzyński Andrzej, Pędziwiatr Michał (2018). Defunctioning ileostomy and mechanical bowel preparation may contribute to development of low anterior resection syndrome. Videosurgery and Other Miniinvasive Techniques.

[48] Celerier B, Denost Q, Van Geluwe B, Pontallier A, Rullier E (2016). The risk of definitive stoma formation at 10 years after low and ultralow anterior resection for rectal cancer. Color Dis.

[49] Nuytens Frederiek, Develtere Dries, Sergeant Gregory, Parmentier Isabelle, D’Hoore André, D’Hondt Mathieu (2018). Perioperative radiotherapy is an independent risk factor for major LARS: a cross-sectional observational study. International Journal of Colorectal Disease.

